# Analysis of Biobanked Serum from a *Mycobacterium avium* subsp *paratuberculosis* Bovine Infection Model Confirms the Remarkable Stability of Circulating miRNA Profiles and Defines a Bovine Serum miRNA Repertoire

**DOI:** 10.1371/journal.pone.0145089

**Published:** 2015-12-16

**Authors:** Ronan G. Shaughnessy, Damien Farrell, Karel Riepema, Douwe Bakker, Stephen V. Gordon

**Affiliations:** 1 UCD School of Veterinary Medicine, University College Dublin, Dublin 4, Ireland; 2 Department of Bacteriology and TSEs, Central Veterinary Institute of Wageningen University, Edelhertweg 15, 8200 AB Lelystad, The Netherlands; 3 Buitenplaats 116, 8212 AM Lelystad, The Netherlands; 4 UCD School of Medicine, University College Dublin, Dublin 4, Ireland; 5 UCD School of Biomolecular and Biomedical Science, University College Dublin, Dublin 4, Ireland; 6 UCD Conway Institute of Biomolecular and Biomedical Science, University College Dublin, Dublin 4, Ireland; Fundació Institut d’Investigació en Ciències de la Salut Germans Trias i Pujol, Universitat Autònoma de Barcelona, SPAIN

## Abstract

Johne’s Disease (JD) is a chronic enteritis of ruminants caused by *Mycobacterium avium* subspecies *paratuberculosis* (MAP). Current disease control strategies are hampered by the lack of sensitive and specific diagnostic modalities. Therefore, novel diagnostic and prognostic tools are needed, and circulating microRNAs (miRNAs) may hold potential in this area. The aims of this study were twofold: (i) to address the stability of miRNA in bovine sera from biobanked samples, and (ii) to assess the potential of miRNAs as biomarkers for JD disease progression. To address these aims we used bovine sera from an experimental MAP infection model that had been stored at -20°C for over a decade, allowing us to also assess the stability of miRNA profiles in biobanked serum samples through comparison with fresh sera. Approximately 100–200 intact miRNAs were identified in each sample with 83 of these being consistently detected across all 57 samples. The miRNA profile of the biobanked sera stored at -20°C for over 10 years was highly similar to the profile of <1 year-old sera stored at -80°C, with an overlap of 73 shared miRNAs. IsomiR analysis also indicated a distinct bovine serum-specific isomiR profile as compared to previously reported bovine macrophage miRNA profiles. To explore the prognostic potential of miRNA profiles cattle defined as seropositive for anti-MAP antibodies (n = 5) were compared against seronegative cattle (n = 7). No significant differential expressed miRNAs were detected at either the early (6 months) or late (43, 46 and 49 months) intervals (FDR≤0.05, fold-change≥1.5) across seropositive or seronegative animals. However, comparing pre-infection sera to the early and late time-points identified increased miR-29a and miR-92b abundance (2-fold) that may be due to blood-cell population changes over time (*P*<0.001). In conclusion our study has demonstrated that bovine circulating miRNAs retain their integrity under long-term sub-optimal storage temperatures opening the way for increased miRNA analyses from biobanked samples for a range of infectious and non-infectious diseases.

## Introduction

Paratuberculosis or Johne’s Disease is chronic enteritis of ruminants caused by *Mycobacterium avium* subspecies *paratuberculosis* (MAP). Exposure to MAP occurs mainly through the faecal-oral route, with maximum susceptibility to infection in the first months of life and resulting in life-long asymptomatic infections for up to 90% of animals [1]. A Th1 cell-mediated immune response is regarded as crucial for maintenance of asymptomatic MAP infection. In contrast, an eventual decline in Th1 responses occurs in the approx. 10% of infected ruminants resulting in the gradual onset of a Th2-biased response, clinical signs, and faecal shedding [2]. Thus on the basis of immunological and other physiological changes, it is possible to distinguish between the different stages of MAP infection via peripheral blood analyses. Immune status monitoring from an early stage of MAP infection may aid in identifying and isolating the specific animals that will develop clinical Johne’s Disease before they commence faecal shedding of the pathogen and become infectious. The ability to monitor the course of infection would facilitate the development of novel disease control strategies based on such an early intervention. However, the commercially available IFN-γ and antibody detection assays for diagnosis of MAP infection suffer with sensitivity and specificity issues, in particular in the first years of infection, thus preventing their use in an early intervention strategy [3]. Novel prognostic biomarkers are therefore warranted that would augment our current tools and allow monitoring of early stages of MAP infection.

Several research groups have investigated global mRNA expression in blood of MAP infected cattle [4–7], although the candidate genes identified have yet to be validated in field studies. Another RNA type that may also hold diagnostic promise are microRNAs (miRNAs). MiRNAs in the circulation have gained prominence as potential biomarkers for a range of disorders and infections, as it is believed that expression profiles of these small regulatory molecules mirror specific abnormalities associated with the development of pathology, immune dysregulation, disease progression, etc. [8]. In contrast to other RNA types, an advantage of circulating miRNAs is their stability, which is believed to be due to the miRNAs being enclosed within vesicles or being associated with protein complexes [9]. Essentially, miRNAs from biofluids such as plasma and serum are highly resistant to freeze thaw, RNAses, room temperature, boiling and pH changes [10,11]. Furthermore, miRNAs can also be amplified from plasma samples stored for five years at -20°C [12] despite the recommended storage temperature being -80°C due to high RNAse activity [13].

The stability of circulating miRNAs opens up the possibility of revisiting biobank samples for maladies such as cancer, cardiovascular disease and infectious diseases, and is particularly useful for samples collected from long-term, time consuming experiments. Johne’s Disease experimental infection models are notable examples of long-term experiments, where 3–5 years are needed to reproduce the clinical stage of infection in cattle and other ruminants [14]. While reverse transcriptase real time quantitative PCR (RT-qPCR) based assays allow relatively facile analysis of miRNA molecules from biofluids the targets must be already known. miRNA-sequencing offers a global approach to characterising circulating miRNA and identifying potential biomarkers [15–17]. It is, however, currently unknown whether aged biobanked samples, stored at suboptimal conditions such as -20°C, would generate artefacts and spurious results with miRNA-sequencing technology. We recently reported successful sequencing of miRNAs from fresh serum volumes (≤ 1ml stored at -80°C) from a six-month MAP bovine infection model [18]. The methods and data analysis pipeline we developed for this latter work were applied to this current study in which we carried out analysis on bovine serum samples that had been stored at -20°C for 10–15 years from a Johne’s Disease model. This enabled us to investigate the stability of biobanked serum with regard to its compatibility with miRNA-seq. Here we report the miRNA profile of sera stored for over a decade at -20C to be strikingly similar to that of fresh sera stored at -80C for less than 1 year, and the use of this data to investigate the potential of miRNA as prognostic biomarkers of Johne’s Disease progression over a 5-year infection model.

## Materials and Methods

### Animals and experimental infection

Project CVI # 804.186.1001 ‘Longitudinal study of paratuberculosis infection in cattle’ was approved by the Animal Experiment Committee (*Dier Experiment Commissie*, DEC) of the Central Veterinary Institute (CVI) and commenced in 1999. Experimental procedures were performed according to Dutch animal welfare legislation (*Wet op de Dierproeven*) and were approved by the competent national authorities.

Twenty Holstein-Friesian female calves were orally infected with 20g of MAP contaminated faeces, three times a week for four weeks during the first months of their life. The inoculum was obtained from a cow showing clinical signs of MAP infection and consistently shedding bacteria. The experimentally infected animals were housed indoors at the facilities of the Central Veterinary Institute (CVI), Lelystad, Netherlands, during the infection experiment. Additionally, for the purposes of a comparative negative set of animals, ten Holstein-Friesian calves from absorbed ELISA negative mothers from the CVI farm were obtained and kept separate from the commercial herd. During the lifetime of the experiment and according to national regulations, feed intake, health, body temperature, drugs administered, and body condition were monitored daily and registered by qualified staff. All animals were kept on a regular diet according to their age and lactation status as would be used in a normal Dutch dairy farm. In addition, all animals were bred at 15 months of age to have them calve and lactating at 24 months of age. Breeding and lactating was continued throughout the lifetime of the experiment as on a commercial farm.

Serum samples were collected from all cattle every two weeks and stored in 1ml aliquots at -20°C for subsequent analyses. Sera were routinely screened at sampling for MAP antibodies using a commercial absorbed ELISA kit (IDvet). S/P cut-off values recommended by the manufacturer were used. Animals with S/P% ratios ≥70% were regarded as positive for MAP antibodies. Rectal samples for faecal culture were taken every two weeks. Samples were cultured on Löwenstein-Jensen media supplemented with 2 μg/ml of mycobactin J (Allied Monitor, Fayette, MO), according to a modified method of Jorgensen [19]. Slants were checked every 4 weeks for growth of MAP for 6 months. If no growth was observed after 6 months of culture, samples were considered to be negative. Colonies were tested for MAP by IS900 PCR [20] and mycobactin dependence. Shedding data was expressed semi-quantitatively in 4 categories: 0 = negative, 1 = 1 cfu/slant, 2 ≤ 50 cfu/slant, 3 ≤100 cfu/slant and 4 ≥100 cfu/slant. When appropriate, or welfare was affected, animals were euthanized using T61 (Intervet International). The experiment was conducted across a 55-month period although some animals perished prior to completion. Ileocaecal-valves, lymph nodes, mesenterial lymph nodes, faecal samples as well as tissue samples from the wall of the ilium, colon and caecum were taken for MAP culture.

### RNA extraction and small RNA sequencing

For miRNA-seq, six experimentally challenged and six control cattle were chosen with serum samples from the 6, 43, 46 and 49 month intervals analysed. These sera samples had been stored at -20°C for over 10 years; miRNA-seq datasets from this infection experiment are therefore referred to as ‘biobanked’ to highlight their storage conditions. These data were compared to previously published miRNA-seq from fresh serum samples, collected from an experimental infection of male animals performed in Ireland and stored at -80°C for less than 1 year (abbreviated to ‘fresh’ samples to differentiate them from the ‘biobanked’ samples) [18].

Using the protocol previously described in [18], small RNAs were extracted from 1 ml serum samples using the miRNeasy Mini Kit (Qiagen). Small RNA yields were determined using Small RNA chips (Agilent Technologies) in conjunction with an Agilent 2100 Bioanalyzer. Of the samples analysed, small RNA concentration ranged from 708 to 2640 pg/μl. Using 5 μl of prepared RNA, libraries were constructed using the TruSeq® Small RNA Sample Preparation Kit (Illumina) with slight deviations from the manufacturer’s protocol described in [18]. The pools were each clustered in two lanes of an Illumina HiSeq 2500 Rapid Run Flow Cell (v1) and sequenced in a SE50bp format using Rapid SBS reagents (Michigan State University RTSF Genomics Core).

### Identification of miRNAs and small RNA fractions

We used the same miRNA discovery algorithms as in our previous study on bovine serum [18]. In brief, miRDeep2 [21] was used to quantify the reads that aligned to known mature miRNAs and for the prediction of potential novel miRNAs. Before submission to miRDeep2, the adapters were trimmed discarding all reads <18nt. Score cut-offs of 4 (signal-to-noise = 5.2) and 0 were used for the novel and known sets respectively. An additional reads-based filter was used to remove the remaining low abundance hits remaining from the miRDeep2 analysis. This filter removed all results with reads present in less than 50% of samples and mean normalised read count < = 200. Raw read counts for each miRNA are normalized to the total number of miRNA reads in each sample by default in miRDeep2. We used the same method for isomiRs.

### IsomiR analysis

sRNAbench [22] (formerly miRanalyzer) was used for isomiR analysis for it’s easy to process output and convenient hierarchical classification scheme (a non-redundant schema). Total miRNA expression levels are computed by summing the canonical form and all its length and sequence variants and are therefore consistent with isomiR profiles. By default, this window is defined as: [Canonical miRNA start–3nt; Canonical miRNA end+5nt]. The results are also highly consistent with the miRDeep2 counts. Post-processing of the sRNAbench results was done with a custom Python script. All samples were aggregated together in one large table for which the count in each sample, mean normalised value and expression as a fraction of its miRNA was calculated for each isomiR. A summary table storing the isomiR count, dominant isomiR and the expression fraction was also calculated.

### Differential expression analysis

EdgeR [23] was used to perform pairwise differential expression (DE) analyses for both conditions (experimentally infected and naturally infected) at each time point. Only the core miRNAs were included in the DE analysis since the remainder would be missing in too many samples (or too low abundance) to be statistically significant. Only samples with FDR <0.05 based on Benjamini and Hochberg multiple testing correction [24] and log fold change >1.5 were considered significant. Python scripts used for post-processing and analysis are available at https://github.com/dmnfarrell/mirnaseq.

## Results

### Animals and experimental infection

**Fig 1:**
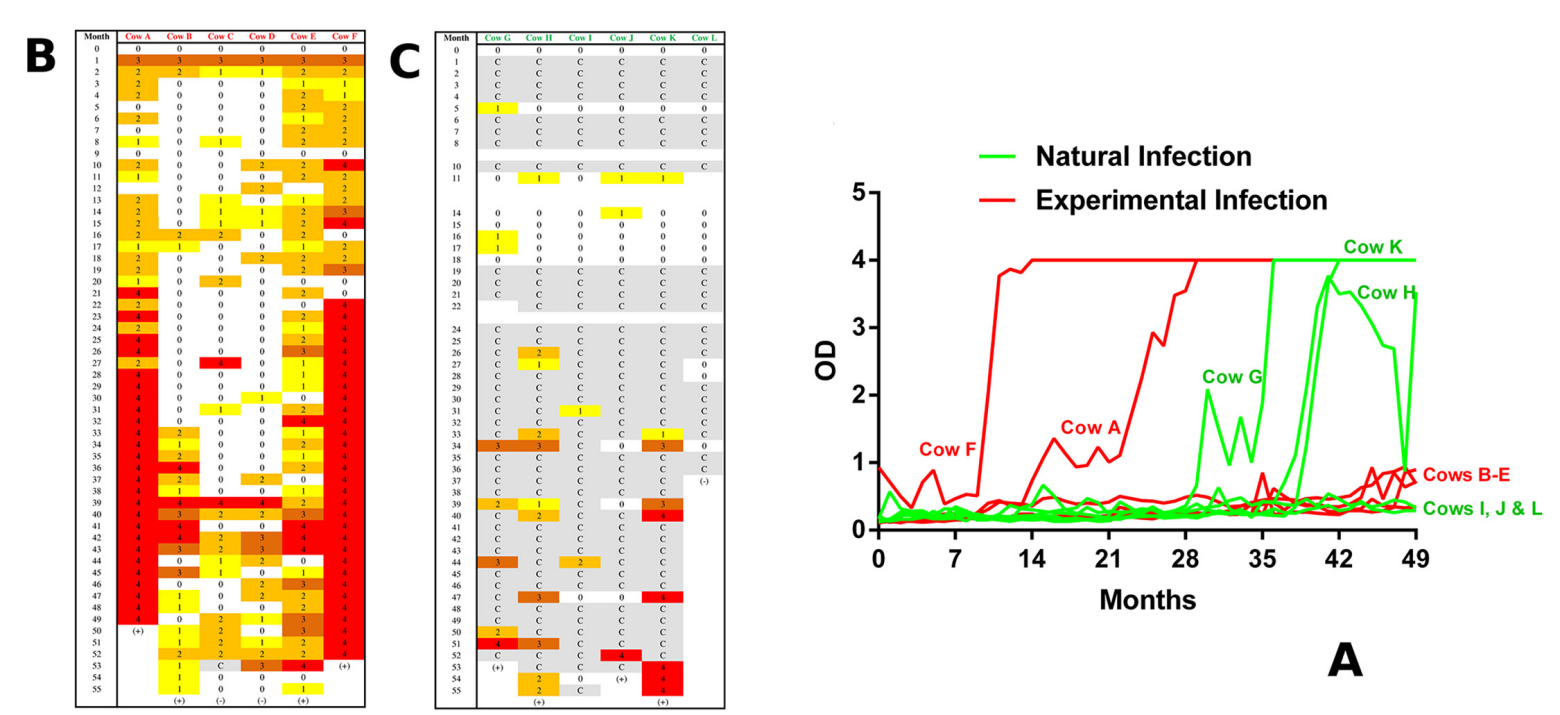
Monitoring of the MAP experimental infection. **(A)** Detection of anti-MAP antibodies in serum via ELISA across 49 months of the time-course. Six individual experimentally infected cattle (in red A-F) and six individual naturally infected cattle (in green G-L) are represented. Faecal culture data for **(B)** experimentally infected and **(C)** naturally infected animals at monthly intervals. 0 = negative, 1 = 1 cfu/ agar slant, 2 = <50 cfu/agar slant, 3 = <100 cfu/agar slant and 4 = >100 cfu agar slant. C = fungal contamination, resulting in an inability to accurately determine MAP cfus.

For the first two months of the CVI experimental infection, MAP was detected in the faeces of all the six experimentally challenged animals utilised in this study (Fig 1B). Faecal shedding subsequently became intermittent for three of the cattle with low rates of MAP detection being evident for 32 months post-challenge. The load and frequency of detectible MAP shedding, however, increased in these three individuals towards the latter stages of the experiment (months 39–52). In contrast, the other three animals became faecal positive within the first year of infection, and MAP shedding remained relatively continuous for the remainder of the experiment. In particular, two of these cattle displayed very high MAP shedding after 22 months (Fig 1B). High levels of MAP antibodies were detected in their sera at this interval and subsequent time-points, suggesting a correlation between the serology and faecal culture diagnostics for these two cattle. There was no clear evidence of seropositivity with the other experimentally infected cattle for the remainder of the time-course (Fig 1A).

Five of the six unchallenged control animals, however, also showed signs of MAP infection throughout the time-course. Despite high levels of fungal contamination rendering many faecal cultures unreadable, low levels of faecal positivity were accurately detected at least once for each animal within the 5–31 month intervals (Fig 1C). In three of these cattle, the frequency of positive culture results and faecal load increased substantially in the latter intervals (month 39–55). These three animals also displayed high levels of MAP antibodies in their sera during the period of high MAP shedding (Fig 1A). While far from an ideal cohort to use for biomarker discovery, we also exploited these sera to assess the potential of miRNA repertoire to categorise MAP infected animals, using the seropositive vs seronegative animals as our two groupings.

### Identification of small RNAs within bovine serum via RNA-seq

**Fig 2:**
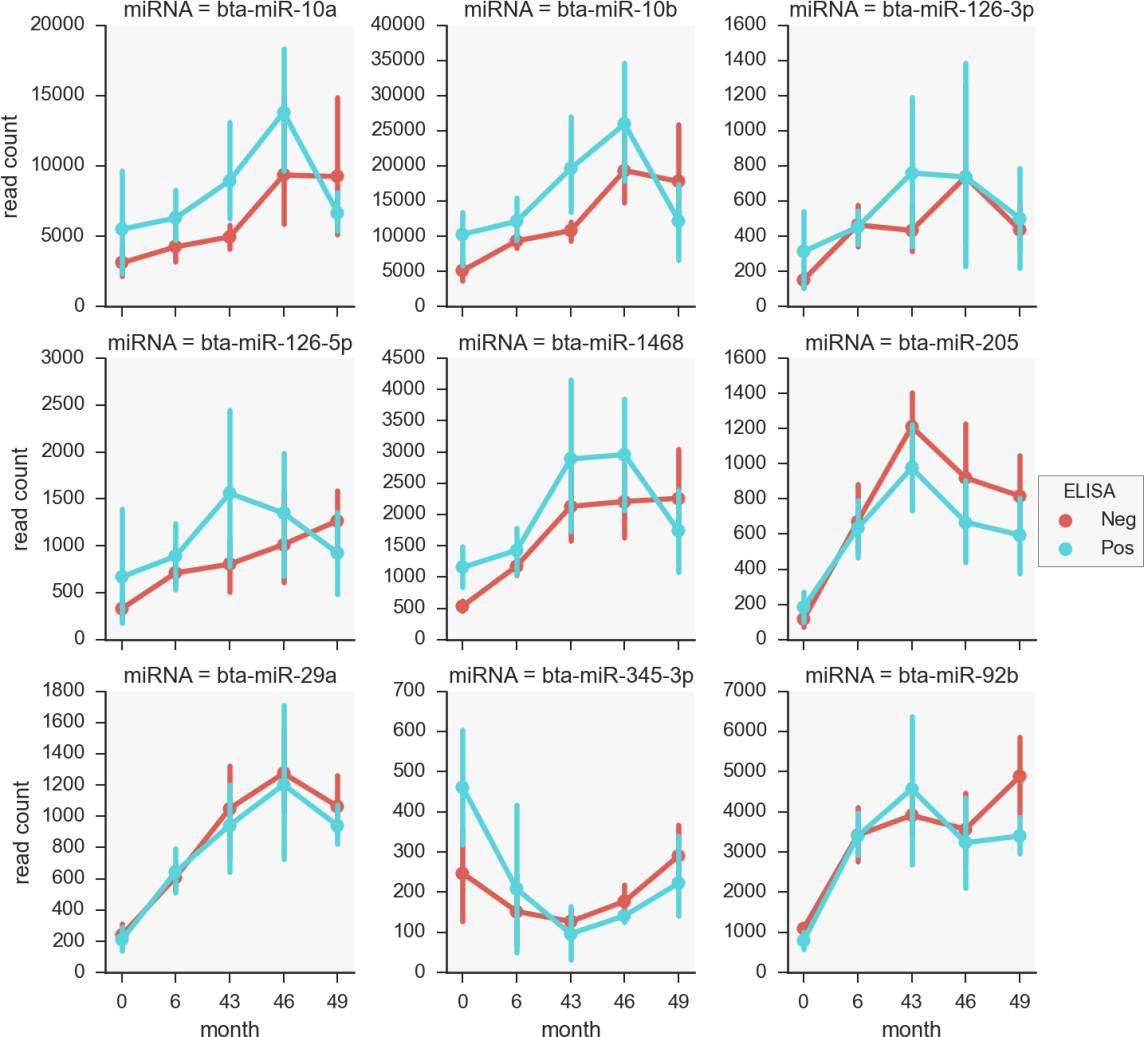
Normalised read variation for differentially abundant miRNA over 5 time points. Normalised reads are shown for distinct miRNA at the 0, 6, 43, 46 and 46-month time points. Note the x axis time interval is not to scale.

We conducted miRNA-seq on 57 sample libraries in total from the CVI biobanked sera, resulting in a total of 311,475,358 reads. Mapping with Bowtie to multiple RNA *Bos taurus* annotations [18] revealed that only a small percentage of reads in each sample (mean 2.7%) mapped to miRNAs. On average, 54% mapped to tRNA, ~10% to other non-miRNA databases while 13% of reads could not be reliably mapped at all (Fig 2A). There were significant variations in small RNA composition between individual samples (S2 Fig). A plot of the typical read length distribution in a sample (S1 Fig) highlights the large amount of degraded small RNA product present in relation to the smaller lengths in the 18–22 range represented by miRNAs.

### Known and novel miRNA profiles

Applying the miRDeep2 algorithm to all 57 samples and applying the score thresholds and other filters as described in the Material and Methods section produced a set of core miRNAs. The core list consisted of 83 known miRNAs (S1 Table) of which the top ten most abundant accounted for 87% of the total. In order of decreasing abundance, the top five were miR-486, miR-423-5p, miR-92a, miR-22-3p and miR-191. The majority of identified miRNAs had homologs in other mammalian species. Only 10% were from the 3 prime strand. There were no significant patterns in global miRNA populations across time points. After filtering out low-scoring and low-frequency results, 5 putative novel miRNAs predicted by miRDeep2 were retained (S2 Table); these were seen at low abundance levels but present consistently in all samples and hence in all 12 animals.

### Comparison of fresh samples to those biobanked at -20°C for over 10 years

We previously generated a miRNA-seq dataset [18] of bovine serum samples from 12 animals that were stored at -80°C for less than 1 year prior to performing miRNA-seq. These represent the miRNA profile of ‘fresh’ serum samples and provide an excellent opportunity for comparative analysis of miRNA profiles and abundance between both fresh and biobanked serum samples. We thus used our miRNA dataset from fresh samples stored at -80°C for less than 1 year as a baseline to reveal any potential storage related effects in the samples from the CVI infection experiment that had been stored for 10–15 years at -20°C. In the following text, serum samples optimally stored at -80°C for less than 1 year are referred to as ‘fresh’ while the 10–15 year CVI samples stored at -20°C are referred to as ‘biobanked’. In addition to this we have chosen for comparison two other external datasets from previous miRNA sequencing studies. To compare within species non serum differences we used a bovine macrophage dataset [25]. For serum non-bovine samples we choose is a set of human serum samples from a study on age-related changes in circulating miRNA [26]. Both sets of read data were downloaded from the sequence read archive [27] and re-analysed in miRDeep2 with identical filters to our own samples.

**Fig 3:**
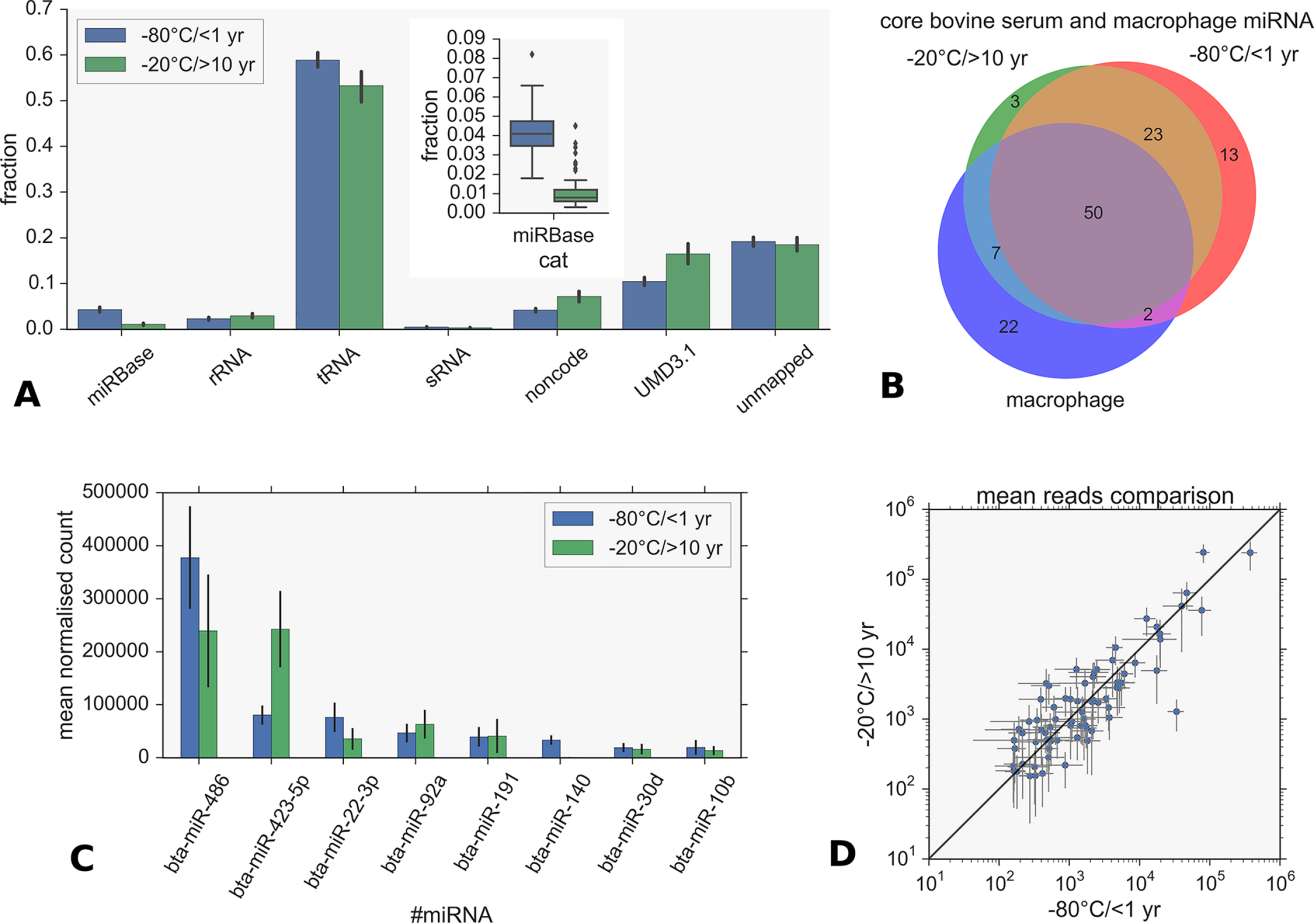
Comparisons between miRNAs from fresh and biobanked serum. A) Fractions of small RNA identified in both fresh (-80°C/<1 year storage) and biobanked (-20^°^C/>10 year storage) according to category using Bowtie mapping. UMD3.1 denotes mappings to the reference genome. miRNA fractions are shown inset in detail. Note these values slightly underestimate the total miRNAs present since they only include miRBase hairpins and not novel content. B) Overlap between fresh (-80°C/<1 year storage) and biobanked (-20°C/>10 year storage) serum core miRNAs and a bovine macrophage dataset. C) Comparison of top 8 miRNAs in fresh (-80°C/<1 year storage) and biobanked (-20°C/>10 year storage) samples shows large differences in the top 2 most abundant miRNAs. Counts are the means of normalised value per sample and error bars show the standard deviation over all n samples. For fresh samples n = 24, for biobanked samples n = 57. D) Correlation between log mean normalised counts for the same miRNA in fresh (-80°C/<1 year storage) and biobanked (-20°C/>10 year storage) datasets. Error bars indicate the variance over all samples of mean read count.

Fractions of small RNA species (Fig 3A) are very similar between the fresh and biobanked samples. Total raw read depth was similar in both cases, but the proportion of reads mapping to miRNA is much higher in the fresh samples (see Fig 3A inset plot) with means of 6.3% (10,684,392 in total) and 2.7% (2,135,533) respectively when all miRDeep2 miRNAs are included and not just miRBase hairpins. The mean reads per sample were 445,183 and 37,465 for the fresh and biobanked samples respectively. The presence of excessive transfer RNA fragments would not account for the difference since there was more tRNA counted in the fresh samples. It is therefore possible that storage related degradation effects may account for this difference. The overlap between core known miRNAs was estimated by setting the same filters on both sets of miRDeep results as described above, using a mean normalised read cut-off of 150. The Venn diagram in Fig 3B shows that the core serum miRNAs are almost identical: this shows that there is a substantial overlap of serum miRNAs across multiple independent experiments as well as across samples and that the serum miRNA population appears distinct from the tissue specific bovine macrophage samples. Non-overlapping miRNAs are detailed in Table 1.

**Table 1:** Differences and similarities in known bovine miRNAs detected between fresh sera (stored at -80°C and <1 year from time of collection) and biobanked sera (stored at -20°C for 10–15 years after collection) following small RNA sequencing.

Fresh serum & Biobanked serum overlap			Fresh serum specific	Biobank serum specific
miR-486	miR-223	miR-660	miR-210	miR-1307
miR-423-5p	miR-222	miR-127	miR-224	miR-17-5p
miR-22-3p	let-7i	miR-125b	miR-532	miR-204
miR-92a	miR-130b	miR-99a-5p	miR-665	miR-2285k
miR-191	miR-16a	miR-92b	miR-141	miR-26b
miR-140	miR-2419-5p	miR-93	miR-365-3p	miR-6119-5p
miR-30d	miR-19b	miR-100	miR-199a-3p	miR-98
miR-10b	miR-326	miR-26a	miR-138	let-7c
miR-192	miR-126-5p	miR-1468	miR-193b	miR-28
miR-423-3p	miR-652	miR-342	miR-361	miR-21-3p
miR-16b	let-7g	151-5p	miR-432	
miR-221	miR-345-3p	miR-769	miR-760-3p	
miR-378	let-7d	miR-197	miR-130a	
miR-151-3p	miR-30b-5p	miR-421	miR-425-5p	
miR-103	miR-301a	let-7f	miR-133a	
miR-21-5p	miR-181a	miR-23b-3p		
miR-107	miR-320a	miR-15a		
miR-215	miR-186	let-7a-5p		
miR-30a-5p	miR-142-5p	miR-205		
miR-2284x	miR-27b	miR-101		
miR-296-3p	miR-30e-5p	miR-328		
miR-29a	miR-148a	miR-126-3p		
miR-27a-3p	miR-150	miR-330		
miR-143	miR-6529a			
miR-181b	miR-10a			
miR-486	miR-223	miR-660	miR-210	miR-1307
miR-423-5p	miR-222	miR-127	miR-224	miR-17-5p
miR-22-3p	let-7i	miR-125b	miR-532	miR-204
miR-92a	miR-130b	miR-99a-5p	miR-665	miR-2285k
miR-191	miR-16a	miR-92b	miR-141	miR-26b
miR-140	miR-2419-5p	miR-93	miR-365-3p	miR-6119-5p
miR-30d	miR-19b	miR-100	miR-199a-3p	miR-98
miR-10b	miR-326	miR-26a	miR-138	let-7c
miR-192	miR-126-5p	miR-1468	miR-193b	miR-28
miR-423-3p	miR-652	miR-342	miR-361	miR-21-3p
miR-16b	let-7g	151-5p	miR-432	
miR-221	miR-345-3p	miR-769	miR-760-3p	
miR-378	let-7d	miR-197	miR-130a	
miR-151-3p	miR-30b-5p	miR-421	miR-425-5p	
miR-103	miR-301a	let-7f	miR-133a	
miR-21-5p	miR-181a	miR-23b-3p		
miR-107	miR-320a	miR-15a		
miR-215	miR-186	let-7a-5p		
miR-30a-5p	miR-142-5p	miR-205		
miR-2284x	miR-27b	miR-101		
miR-296-3p	miR-30e-5p	miR-328		
miR-29a	miR-148a	miR-126-3p		
miR-27a-3p	miR-150	miR-330		
miR-143	miR-6529a			
miR-181b	miR-10a			

In terms of abundance profiles we found a surprisingly high degree of consistency (correlation co-efficient of 0.87) of log-transformed mean normalised read counts for the same miRNA between the fresh and biobanked samples. For comparison the mean correlation between biological replicates (individual animals at the same time point and infection state) in the biobanked samples was ~0.92. Fig 3C shows the total read counts compared for the top 10 abundant miRNAs and Fig 3D shows the correlation in read counts for all miRNAs present in both sets with error bars indicating the standard deviation over all samples. Despite differences in animals, age of samples and experimental conditions, read counts between experiments are largely within the total sample-wide variation of each dataset. There is no indication from these results that storage time effects have degraded the miRNA data in any systematic way apart from the differences in read depth.

### IsomiR analysis

As with most previous studies, our differential expression analysis considers mature miRNA levels; all isoforms, or isomiRs, of the mature sequence are counted together. It is however possible that the current concept of the mature miRNA is too limited and that considering isomiR expression profiles individually could provide improved markers. Recent work [28] in humans indicated certain isoforms vary between gender and sub populations. This may have an impact on biomarker analysis using only mature forms. For example, changes in a particular isomiR might be significant but masked if only considered as a component of the mature product.

**Fig 4:**
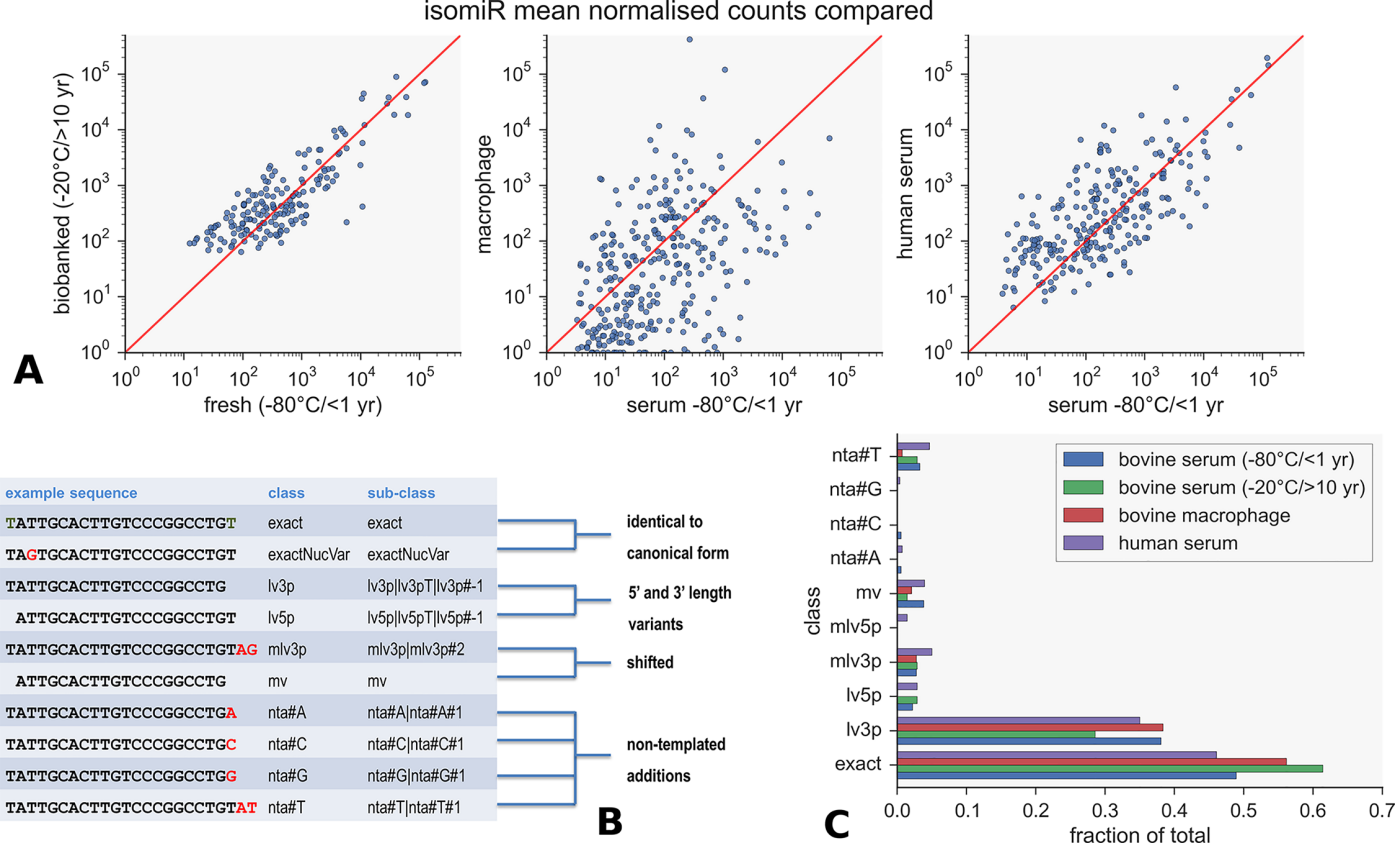
IsomiR abundances and variant representation in different datasets. A) IsomiR normalised counts compared between 1) fresh (-80°C/<1 year storage) and biobanked (-20°C/>10 year storage) bovine serum, 2) serum and macrophage (both bovine) and 3) bovine and human serum. B) Classification scheme for isomiRs using sRNAbench is hierarchical. *exactNucVar* means single nucleotide changes to the canonical sequence, most probably due to sequencing errors. *mv* indicates shifted sequences. non-templated addition is enzymatically addition of a nucleotide to the 3’ end and is given priority by sRNAbench since these changes may be of biological relevance. C) Plot shows the counts of dominant isomiRs categorised by class. The general trend of dominance is the same across all datasets, including non-serum.

Using sRNAbench, we obtained the abundance profiles of isomiRs across both fresh and biobanked samples. In the following we use sRNAbench nomenclature to describe the isomiR classes, as explained in Fig 4B. The results were filtered to remove all low abundance isomiRs (total reads<10 across all samples and present in less than 50% of samples) that could be attributable to sequencing or alignment artefacts. As described in other studies [29] for many miRNAs the dominant isomiR was different to the reference (canonical) miRBase mature sequence. For our fresh samples the dominant and canonical matched in 49% of cases, while for the biobanked samples this value was 61%. Only a small fraction of miRNAs did not have more than 1 isomiR. Also notable was that the fraction of each isomiR (as a proportion of total reads for that miRNA) was somewhat correlated between fresh and biobanked samples, though much less so at low fractions (S4 Fig). That is, the same isomiR is generally highly represented across both sets of serum data. Mean abundance profiles were compared between -80°C/<1yr and -20°C/>10yr data sera and between bovine (-80°C/<1yr) and human serum and are shown in Fig 4A. The clear correlations between bovine serum abundances (correlation coefficient = 0.82, log_2_ scale) and even between bovine and human serum (cc = 0.69) contrast with the relationship between bovine serum and macrophage bovine isomiRs (cc = 0.43).

**Fig 5:**
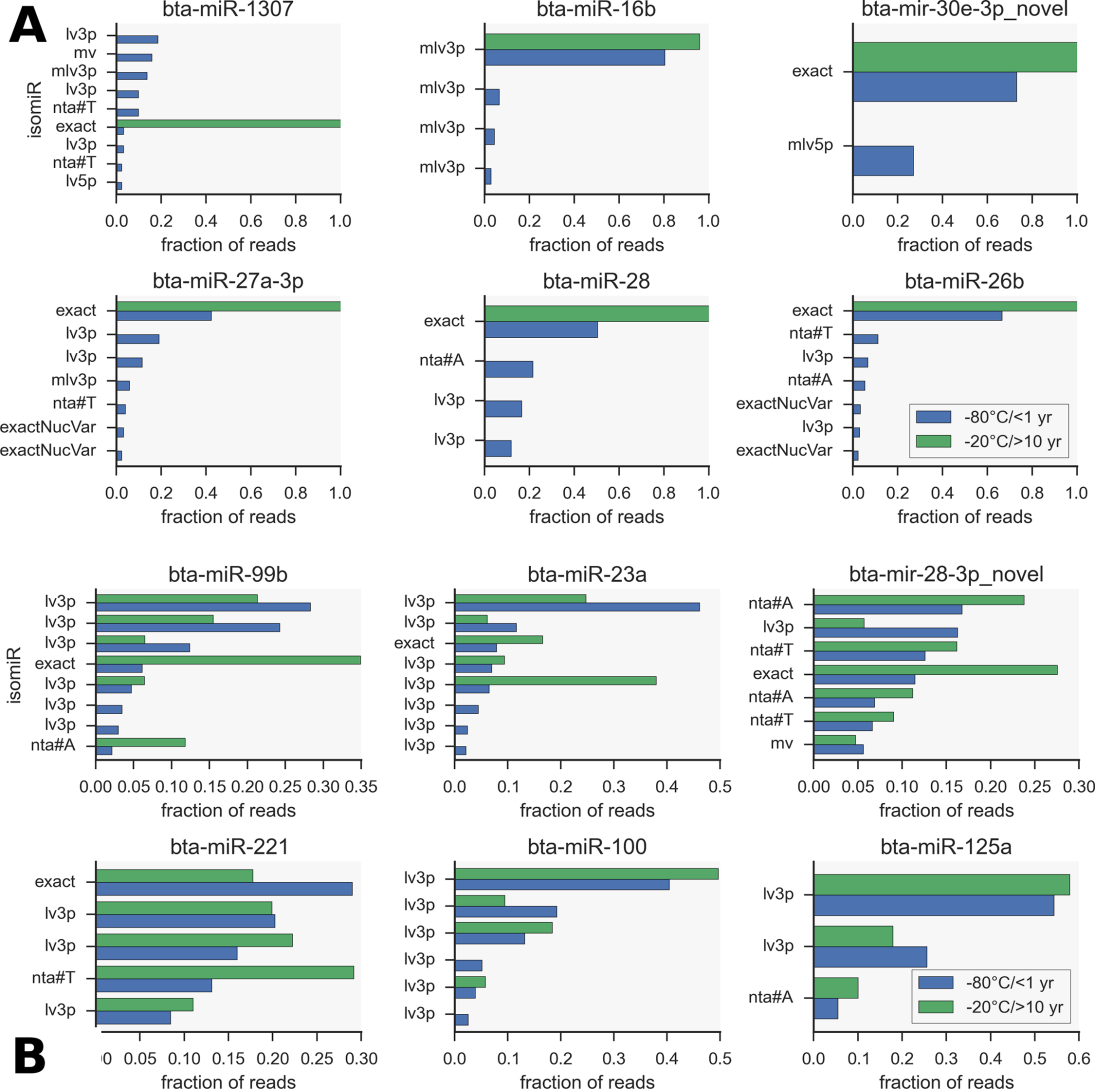
isomiR abundance patterns compared between fresh and biobanked serum. A) Relative abundances of isomiRs with a single dominant form in the fresh (-80°C/<1 year storage) dataset but not in the biobanked (-20°C/>10 year storage) data. Note that many of the low abundance forms are not present in the biobanked data (green bars). B) Relative abundances (shown as fraction of total) of some miRNAs with multiple sub-dominant isomiRs compared between fresh and biobanked year samples.

IsomiR relative abundance distributions followed a pattern seen in previous studies [30]: those miRNAs with one strong predominant isomiR and on the other end of the spectrum are miRNAs with multiple sub-dominant variants. The canonical form is often not the most abundant or even present. This is illustrated in S5 Fig that shows the miRNAs binned according to the number of isomiRs that account for at least 5% of abundance thus reflecting the number of dominant forms. Those with a value of 1 are single dominant isomiRs and are in the minority. As can be seen 2–3 dominant isomiRs is the most common case. Datasets from both fresh and biobanked samples are shown and have similar distributions; there is a consistent pattern of isomiR dominance between the 2 datasets with the same dominant form 87% of the time. The breakdown of dominant isomiRs by class is shown in Fig 4C, indicating the same trend of class representation even across the serum and macrophage datasets. Fig 5A and 5B show relative (fraction of total) abundances for the most single dominant and sub-dominant cases respectively in both datasets. Fig 5A highlights cases where there is a single dominant isomiR in the biobanked dataset, but in the data from the fresh sera more isomiRs are seen. This is generally seen for lower abundance miRNAs where the lower read depth in the fresh samples means these rare isomiRs are simply not sampled (all variants with less than 1% of reads within the miRNA were removed for this analysis). bta-miR-28 and bta-miR-26b are examples; more single isomiRs are seen in the data from the fresh samples for this reason (S5 Fig). In some cases the differences are more likely to be biological where the miRNA is simply much less abundant in the other dataset, bta-miR-1307 and bta-miR-16b being good examples. Single dominant isomiRs almost always correspond to the canonical form as presumably they will appear unambiguously in sequencing data.

### isomiR differential expression

**Fig 6:**
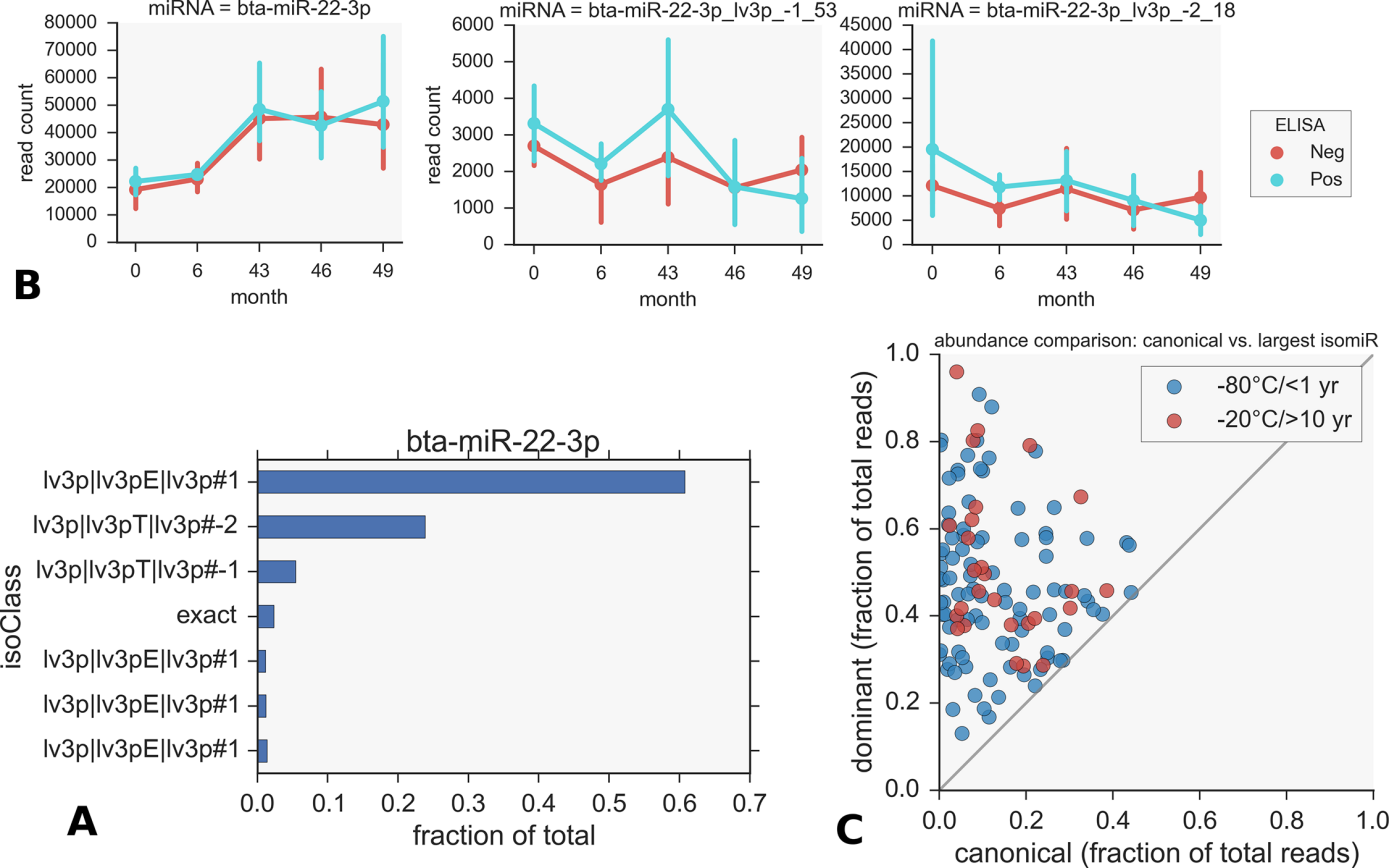
Canonical and dominant isomiR differences. A) bta-miR-22-3p total abundance profile over time (left) and for 2 isomiRs (right), both are shorter 3’ variants. Analysing these isomiRs individually allows them to be detected as being differentially expressed over time. B) bta-miR-22-3p isomiR relative abundances. The canonical form, labelled *exact*, is only fourth most abundant. C) The canonical and dominant isomiRs differ in >50% of cases. Shown in the plot are the corresponding percentages between the 2 isoforms, when different. (By definition *dominant* means highest percentage of reads). For points in the top left of the plots, the actual canonical form is insignificant. Note the lower number of points for the 10–15 year (CVI) dataset.

It is clear that each isomiR will have its own unique expression profile. Given the frequent presence of multiple dominant forms, an analysis of the isomiR differential expression on an individual basis could provide a different result from the total miRNA abundances. We therefore did the same analysis described above but for individual isomiRs. For the most part the results are as expected, with the analysis revealing dominant forms of the same miRNAs that are already indicated in Fig 2. However 2 isomiRs of bta-miR-22-3p appear as differentially expressed in this analysis. These are the second and third most abundant forms, the top 3 isomiRs being 3’ length variants as shown in Fig 6A. In this case it is clear that simply considering the total abundance level (all isomiRs summed) could conceal a distinctive isomiR signature. The total abundance profile of bta-miR-22-3p and 2 isomiR profiles are shown for comparison in Fig 6B: note the much smaller read counts for the isomiRs however. We found the canonical and dominant isomiRs to differ in over 50% of cases. Most of these isomiRs are 3 prime length variants; examples are listed in Table 2. The correlation between relative abundances in these cases are shown in Fig 6C for datasets from both fresh and biobanked samples. This indicates how often the supposed canonical form makes up only a fraction of the reads compared to the actual dominant form.

**Table 2:** miRNAs where dominant isomiR is at least 3 times more abundant than the canonical form.

name	Canonical sequence	variant	Can. reads	Total reads	Can. frac	Dom. frac
bta-miR-16b	TAGCAGCACGTAAATATTGGC	mlv3p	69	1706	0.04	0.96
bta-miR-30c	TGTAAACATCCTACACTCTCAGC	mlv3p	273	3073	0.09	0.83
bta-miR-103	AGCAGCATTGTACAGGGCTATGA	lv3p	719	9198	0.08	0.80
bta-miR-23b-3p	ATCACATTGCCAGGGATTACCAC	lv3p	122	585	0.21	0.79
bta-miR-326	CCTCTGGGCCCTTCCTCCAG	nta#T	91	1086	0.08	0.65
bta-miR-22-3p	AAGCTGCCAGTTGAAGAACTG	lv3p	1704	72239	0.02	0.61
bta-miR-125a	TCCCTGAGACCCTTTAACCTGTG	lv3p	153	2305	0.07	0.58
bta-miR-125b	TCCCTGAGACCCTAACTTGTGA	lv3p	142	1466	0.10	0.51
bta-miR-140	TACCACAGGGTAGAACCACGGA	mv	99	1235	0.08	0.50
bta-miR-100	AACCCGTAGATCCGAACTTGTG	lv3p	233	2235	0.10	0.50
bta-miR-93	CAAAGTGCTGTTCGTGCAGGTA	lv3p	95	1046	0.09	0.46
bta-miR-342	TCTCACACAGAAATCGCACCCATCT	lv3p	126	993	0.13	0.44
bta-miR-10b	TACCCTGTAGAACCGAATTTGTG	lv3p	1063	20986	0.05	0.42
bta-miR-423-3p	AAGCTCGGTCTGAGGCCCCTCAGT	lv5p	1596	39823	0.04	0.40
bta-miR-181a	AACATTCAACGCTGTCGGTGAGTT	lv3p	2436	41895	0.06	0.38
bta-miR-10a	TACCCTGTAGATCCGAATTTGTG	lv3p	474	11223	0.04	0.37

In many cases the lower number of counts when considering isomiRs will not provide sufficient statistical power for detecting differential expression, or will not be biologically relevant and isomiR analysis will not be appropriate. Noise and quantization effects at low abundances become significant [20] and sequencing biases such as GC composition [19] seen in isoforms for RNA-seq data might also be important for miRNA. Therefore caution is needed, but such an approach may give a more coarse grained way to analyse differential expression as has been recently shown [31].

### Differential miRNA expression

As stated above, evidence of MAP infection in many of the control animals rendered these sera samples far from ideal for use as comparators against infected animals for biomarker studies. However, given the timescale and expense of MAP infection models, plus the need to ensure maximal use of biobanked samples from animal experimental studies (in keeping with the principles of the 3Rs), we chose to pursue a number of pairwise comparisons to investigate differential miRNA expression between seropositive and seronegative animals.

With the obvious complexities in defining disease progression in MAP, we reasoned that the simplest initial approach would be to compare the five MAP-antibody-positive animals (two experimentally infected and three naturally infected) against the remaining seven seronegative animals, as this grouping represented a clear shift in the adaptive immune response. However, using a combined FDR of <0.05 and a fold-change of 1.5 as cut-offs for significance, no differentially expressed miRNAs were identified at either the early (6 months) or late (43, 46 and 49 months) intervals.

In contrast, within group comparisons yielded some differentially expressed miRNAs. A comparison of the cattle that became seropositive against their respective time point 0 samples (pre-bleeds) identified significant miR-29a and miR-92b increases at the early interval (6 months). These two miRNAs were also significantly increased at the late interval (43, 46 and 49 months amalgamated) and accompanied by miR-205 upregulation (Table 3). However, these differences in miRNA abundance appeared to be independent of infection status, as the seronegative animals had a similar expression pattern: miR-92b was upregulated at the early interval and remained increased in the late interval together with miR-205 and miR-29a (Table 3). These circulating miRNA profile changes may potentially represent an ageing effect, but we can also not exclude that they may also reflect mycobacterial infection as both groups showed evidence of MAP infection, an infection which most usually happens in the first months of life.

**Table 3:** Differential miRNA expression between the pre-bleed intervals (time-point 0) and the latter intervals, early (6 months) and late (43, 46 and 49 months amalgamated), within each of the sero-positive (n = 5) and seronegative (n = 7) groups (FDR ≤0.05, fold-change ≥1.5).

Gene	Log_2_ Fold Change	P value	FDR
**Sero-positive—Early**			
bta-miR-92b	2.23	9.82×10^−6^	0.00073
bta-miR-29a	1.72	0.00101	0.03739
**Sero-positive—Late**			
bta-miR-29a	2.24	0.00020	0.00757
bta-miR-92b	2.08	0.00074	0.01100
bta-miR-205	1.68	0.00294	0.02911
bta-miR-345-3p	-1.59	0.00012	0.00757
**Sero-negative—Early**			
bta-miR-205	2.05	3.33E-05	0.00086
bta-miR-92b	1.55	1.42E-05	0.00086
**Sero-negative—Late**			
bta-miR-205	2.62	8.42×10^−9^	2.08×10^−7^
bta-miR-29a	2.19	1.52×10^−14^	1.13×10^−12^
bta-miR-1468	1.92	1.00×10^−9^	3.72×10^−8^
bta-miR-92b	1.75	2.44×10^−8^	4.52×10^−7^
bta-miR-126-3p	1.63	3.12×10^−5^	0.00033
bta-miR-126-5p	1.59	4.97×10^−6^	7.36×10^−5^

Subsequently for each individual time-point, the reads counts of the top six differentially expressed miRNAs identified during the pairwise comparisons were plotted to gain further insight into their expression profiles (Fig 2). For the majority of the intervals, there were similar expression patterns between the seropositive and seronegative groups for miR-29a, miR-101, miR-205, miR-92b, miR-345-3p and miR-1468. Another key observation was that the read counts were highly variable between animals, particularly for the latter time-points of both groups (43, 46 and 49 months). This may partially account for the lack of differentially expressed miRNAs between the ELISA positive and negative groups.

## Discussion

Circulating miRNAs have become a popular aspect of small RNA research, largely due their potential as prognostic or diagnostic markers for various pathologies [8,32]. However, miRNAs are somewhat poorly understood as much remains to be defined about their origins and precise functions [33,34], and whether or not they are predominantly restricted to vesicles [35,36]. Furthermore, their inherent stability has yet to be explored with RNA sequencing technology. In this study, we had the opportunity to use miRNA-seq to compare miRNA repertoires in biobanked bovine serum from MAP experimental infections that had been stored at -20°C for 10–15 years or< 1 year at -80°C, and to explore the potential of miRNA profiling to monitor the course of disease progression during infection of cattle with MAP

The small RNA profile of biobanked 10–15 year serum frozen at -20°C was remarkably similar to that from fresh serum stored for <1 year at -80C, with comparable percentages of sequence reads mapped to rRNA, sRNA, tRNA and miRNA databases. Over half of the reads represented tRNA fragments ranging from 18–35 bp (see S1 Fig) in each dataset and the assumption would be that their high abundance is due to random degradation of larger tRNAs (76–90 bp) due to the high RNAse activity in blood [37]. One might thus expect their percentage to be higher in the biobanked samples, particularly due to their long-term storage at -20°C, yet tRNA abundance was surprisingly higher in the fresh samples. Many of these short tRNAs may instead be present to fulfil a specific regulatory role as some of these molecules have been shown to function analogously to miRNAs [38]. In comparison to the fresh samples, less reads from the biobanked samples mapped to the archetypal miRNAs in miRBase. This might reflect a deterioration of non-circulating miRNAs over time. During biofluid preparations, it is challenging to completely remove contaminating cellular miRNAs arising from blood cells but these RNAs would not typically be protected from the action of RNAses [39,40]. Thus, the miRNAs remaining in the biobanked samples may represent ‘true’ circulating miRNAs after degradation of contaminating cellular miRNA.

We identified a core list of 83 known bovine miRNAs that were stably expressed across the majority of biobanked samples. Over 70% of these miRNA genes are orthologs to characterised human circulating miRNAs [41], suggesting that most of the identified 21–25 nt molecules are of a cell-free nature. Surprisingly, there was a high degree of overlap (73 miRNAs in common) between the serum miRNAs detected in the fresh and biobanked samples, even though the datasets originated from male and female cattle respectively. Thus, a core bovine serum-specific miRNA repertoire is evident between animals. Of the less than 20 miRNAs that did not overlap each of the datasets, these were at low abundance in the opposing sample-set and not in the ‘core’ set. Exosomal miRNAs from human plasma stored at -20°C for five years are known to be stable [12]. Our data further suggests that circulating miRNA stability is not substantially affected by long term storage -20°C, and that the samples can be subsequently used with miRNA-sequencing.

The majority of circulating miRNA studies to date have focused on cancer diagnostics [8] but recently microbial infections have been investigated, particularly mycobacterial infections [17,42–45]. We recently investigated miRNA expression in bovine serum from a six month MAP bovine infection experiment [18], a time point that is much too early to capture the progression of MAP infection. The CVI experimentally infected animals used in the current study, however, have been sampled across a five-year period and encompassing the range of presentation from silent to subclinical Johne’s Disease stages. This provided a unique opportunity to assess whether the serological miRNA expression profile changes in alignment with infection progression.

Upon post-mortem, MAP was detected in gut-associated lymphoid tissue (GALT) from experimentally challenged cattle indicating successful infection. However, most of the animals intended to serve as age-matched negative controls eventually also showed signs of infection, as faecal shedding of MAP became apparent. These ‘natural’ infections are a consequence of the MAP prevalence in dairy herds in the Netherlands, estimated to be as high as 71% [46]. Accordingly, animals were regrouped on the basis of the ELISA and faecal shedding data. A detectable humoral immune response to MAP is indicative of progression towards clinical Johne’s and is typically accompanied by increased faecal shedding of the microbe [1,2]. In agreement, the two experimentally infected cattle that had detectible MAP-antibodies were also the two highest faecal shedders. Three naturally infected cattle were ELISA positive, and these were also the highest shedders within the control groups. In this regard, comparing the differential miRNA abundance in age-matched ELISA-positive to ELISA-negative animals was deemed the most prudent approach.

Utilising stringent FDR and fold-change cut-offs to minimise false-positives, we observed no significant differential miRNA expression between the ELISA-positive/high shedders and ELISA-negative/low shedders at any time-interval. The lack of differential expression might be a consequence of both groups of cattle being MAP-positive, making it difficult to identify a signal for infection. Differences were, however, apparent in the comparisons of time-point 0 to the subsequent intervals within each group. Interestingly, miR-29a was increased in both the sero-positives and sero-negatives. With regard to *M*. *tuberculosis* studies, this miRNA has been identified as a serological biomarker [43] and was shown to be differentially expressed in leukocytes during TB infection [47,48]. MiR-29a has also been reported to be increased in human macrophages in response to *M*. *avium* subsp. *hominissuis* [49]. The miR-29a increase we observed, thus, could be a response to infection but there is also the possibility that this is an age-induced effect due to calves maturing. We recently reported miR-205 and miR-92b increases in the sera of healthy MAP-free calves after six months of development [18] and in this study, both these miRNAs had almost identical patterns of expression at the six month interval. These expression changes may reflect changes in the peripheral blood cell population. In neonatal calves, γδ T-cell numbers gradually decrease while conventional T cells and B lymphocytes increase over time [50,51]. Furthermore, orthologs of both these miRNAs have been detected in human leukocytes [52] so there is the possibility of a similar expression profile in bovine leukocytes. Regardless of the precise origins of this age-related miRNA increase, data presented here and in our recent publication [18] illustrates the importance of using age-matched controls in accurately measuring differential miRNA expression.

IsomiR discovery has become a feature of many miRNA-seq experiments and commonly the dominant miRNA variant is not the canonical mirBase sequence [53]. We have therefore examined in some detail the isomiR profiles of our fresh and biobanked bovine serum samples. Within the 57 biobanked serum samples, we identified approximately 16 miRNAs where the dominant isomiR was at least three times more abundant than the canonical form. The same patterns are seen in fresh sera. The mean isomiR abundances in the biobanked samples was more strongly correlated to isomiR abundances from the fresh samples, and even to human serum, than to cellular-specific bovine macrophage samples. This shows that there is a consistent and serum specific isomiR profile and that isomiR profiles are consistent across samples. The mechanisms of isomiR production are not yet fully understood, but it is clear that these are not simply degradation products.

Shared dominant isomiRs appear to be very common. For multiple sub-dominant cases it is not surprising that the canonical form is often misidentified as the miRBase annotation may rely on data from a single experiment where this form was most abundant. Difference in depth between experiments affects the ability to discern low abundance forms as seen when we do a direct comparison between corresponding isomiRs in the data from fresh and biobanked samples. No other systematic differences, such as in isomiR class distribution, were seen. Examination of isomiR differential expression yielded results consistent with the total abundances. Two variants of bta-miR-22-3p were also shown to be altered in the early time points. It is clear from this work that consideration of isomiRs in both differential expressions analysis and in creating primers for PCR quantification could have an important bearing on results.

The 5’ miRNA variants (lv5p) have been shown to also function as regulators of gene expression and may have different targets to their canonical counterparts [53–55]. The majority of detected isomiRs were, however, 3’ variants and their functions are poorly defined [29,56]. miRNA integrity may be an aspect of these isomiRs, as 3’ variants arising from non-template nucleotide additions have greater stability [57,58]. This unfortunately does not apply to our data though as the majority of 3’ variants were shorter than the canonical equivalents and there was very little evidence of adenylation or other terminal non-template nucleotide additions. Instead, the varying miRNA lengths may have been randomly produced by imperfect Dicer processing [59].

Analyses of our dataset from fresh sera showed significant differences in the two most abundant miRNAs, bta-miR-486 and bta-miR-423-5p. As shown in Fig 3C and 3D, the sample variation usually accounts for much of the difference in normalised counts but for these two miRNAs there is a large discrepancy. The over representation of bta-miR-486 in the fresh samples is the cause of this and raises the issue of correct normalisation procedures for miRNA [60]. We adopted the normalization method of miRDeep2 by using total mapped miRNA reads for all analysis and no other normalizations where performed. Endogenous reference genes for normalisation need to be steadily expressed in all samples at the same expression level and we do not find these requirements are satisfied for any miRNA in our data. An additional caveat is that biases introduced during library preparation may skew the representation of particular miRNAs that in turn may have downstream implications for data normalisation [60]. Our methods were sufficient for general comparisons between independent experiments but biological conclusions from such results must be treated with caution; this also applies to isomiR abundances.

In summary, we have used miRNA-sequencing technology to further confirm the remarkable stability of circulating miRNAs. We have revealed that the miRNA repertoire and expression profile in bovine serum stored at -20°C for a 10–15 year period is almost identical to that of <1-year-old bovine serum optimally-stored at -80°C. Given the high prevalence of MAP infection in cattle herds in many parts of the world, detection of infection *per se* as part of a test and cull eradication scheme is not sufficient. The ideal diagnostic would identify infected animals progressing to clinical disease and high shedding status for removal, hence reducing transmission within an infected herd by early culling of animals identified as a potential risk. While a miRNA biosignature of MAP infection was not revealed, it is possible that the absence of clinical animals in our MAP infected animals may have limited our study’s sensitivity to detect miRNA changes; future analyses will therefore focus on miRNA profiles in clinical vs. subclinical and uninfected animals.

## Supporting Information

S1 Fig Read length distribution for a representative sample after adapter trimming. The large numbers of reads>30 represent tRNA degradation product and the small peaks around 20 reads represent the miRNA content. Reads <18nt were removed for later miRNA analysis.
(PNG)

S2 Fig Bar chart of small RNA categories mapped to each sample by percentage. The samples are sorted by their timepoint (month) and ELISA status.
(PNG)

S3 Fig Proportions of isomiR class frequencies in all known miRNAs. Labels for isomiR classes are using the sRNAbench hierarchical scheme.
(PNG)

S4 Fig IsomiR relative abundance (fraction of the total miRNA reads) compared between 1) fresh (-80°C/<1 year storage) and biobanked (-20°C/>10 year storage) bovine serum, 2) serum and macrophage (both bovine) and 3) bovine and human serum. (PNG)

S5 Fig Distributions of miRNAs binned by counts of dominant isomiR for both fresh (-80°C/<1 year storage) and biobanked (-20°C/>10 year storage) datasets, in this case defined as those isomiRs >5% of the total reads for that miRNA. 2–4 dominant variants is most typical. (PNG)

S6 Fig Plot showing the relationship between no. of dominant isomiRs and abundance. (PNG)

S1 Table Known miRNAs specified by miRDeep2 that meet the filtering criteria described in the paper. (CSV)

S2 Table Novel miRNAs identified with miRDeep2. (CSV)
