# Correction: Analysis of Biobanked Serum from a *Mycobacterium avium* subsp *paratuberculosis* Bovine Infection Model Confirms the Remarkable Stability of Circulating miRNA Profiles and Defines a Bovine Serum miRNA Repertoire

**DOI:** 10.1371/journal.pone.0147355

**Published:** 2016-01-19

**Authors:** The PLOS ONE Staff

There is an error in the third sentence of the Identification of small RNAs within bovine serum via RNA-seq section of the Results. The correct sentence is: On average, 54% mapped to tRNA, ~10% to other non-miRNA databases while 13% of reads could not be reliably mapped at all (Fig 3A).

There are errors in Table 1. The publisher apologizes for these errors. Please see the corrected Table 1 here.

**Table 1:** Differences and similarities in known bovine miRNAs detected between fresh sera (stored at -80°C and <1 year from time of collection) and biobanked sera (stored at -20°C for 10–15 years after collection) following small RNA sequencing.

Fresh serum & Biobanked serum overlap			Fresh serum specific	Biobank serum specific
miR-486	miR-223	miR-660	miR-210	miR-1307
miR-423-5p	miR-222	miR-127	miR-224	miR-17-5p
miR-22-3p	let-7i	miR-125b	miR-532	miR-204
miR-92a	miR-130b	miR-99a-5p	miR-665	miR-2285k
miR-191	miR-16a	miR-92b	miR-141	miR-26b
miR-140	miR-2419-5p	miR-93	miR-365-3p	miR-6119-5p
miR-30d	miR-19b	miR-100	miR-199a-3p	miR-98
miR-10b	miR-326	miR-26a	miR-138	let-7c
miR-192	miR-126-5p	miR-1468	miR-193b	miR-28
miR-423-3p	miR-652	miR-342	miR-361	miR-21-3p
miR-16b	let-7g	151-5p	miR-432	
miR-221	miR-345-3p	miR-769	miR-760-3p	
miR-378	let-7d	miR-197	miR-130a	
miR-151-3p	miR-30b-5p	miR-421	miR-425-5p	
miR-103	miR-301a	let-7f	miR-133a	
miR-21-5p	miR-181a	miR-23b-3p		
miR-107	miR-320a	miR-15a		
miR-215	miR-186	let-7a-5p		
miR-30a-5p	miR-142-5p	miR-205		
miR-2284x	miR-27b	miR-101		
miR-296-3p	miR-30e-5p	miR-328		
miR-29a	miR-148a	miR-126-3p		
miR-27a-3p	miR-150	miR-330		
miR-143	miR-6529a			
miR-181b	miR-10a			
