# Supplementary figures and images for: Analysis of Biobanked Serum from a Mycobacterium avium subsp paratuberculosis Bovine Infection Model Confirms the Remarkable Stability of Circulating miRNA Profiles and Defines a Bovine Serum miRNA Repertoire
Source: PLoS One. 2015 Dec 16;10(12):e0145089. doi: 10.1371/journal.pone.0145089 (PMC4682966; doi:10.1371/journal.pone.0145089)

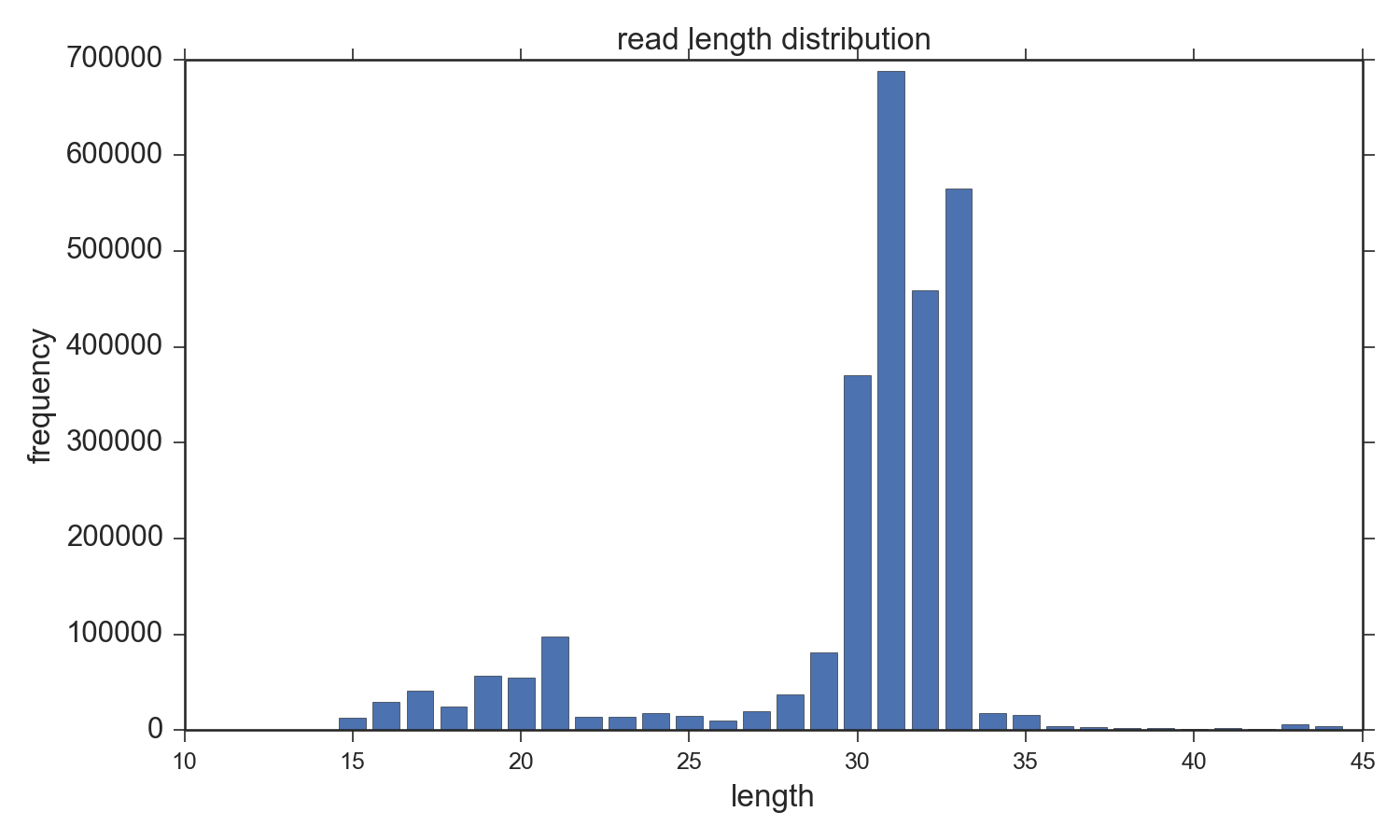

Supplement: S1 Fig — The large numbers of reads>30 represent tRNA degradation product and the small peaks around 20 reads represent the miRNA content. Reads <18nt were removed for later miRNA analysis. (PNG) [file pone.0145089.s001.png]

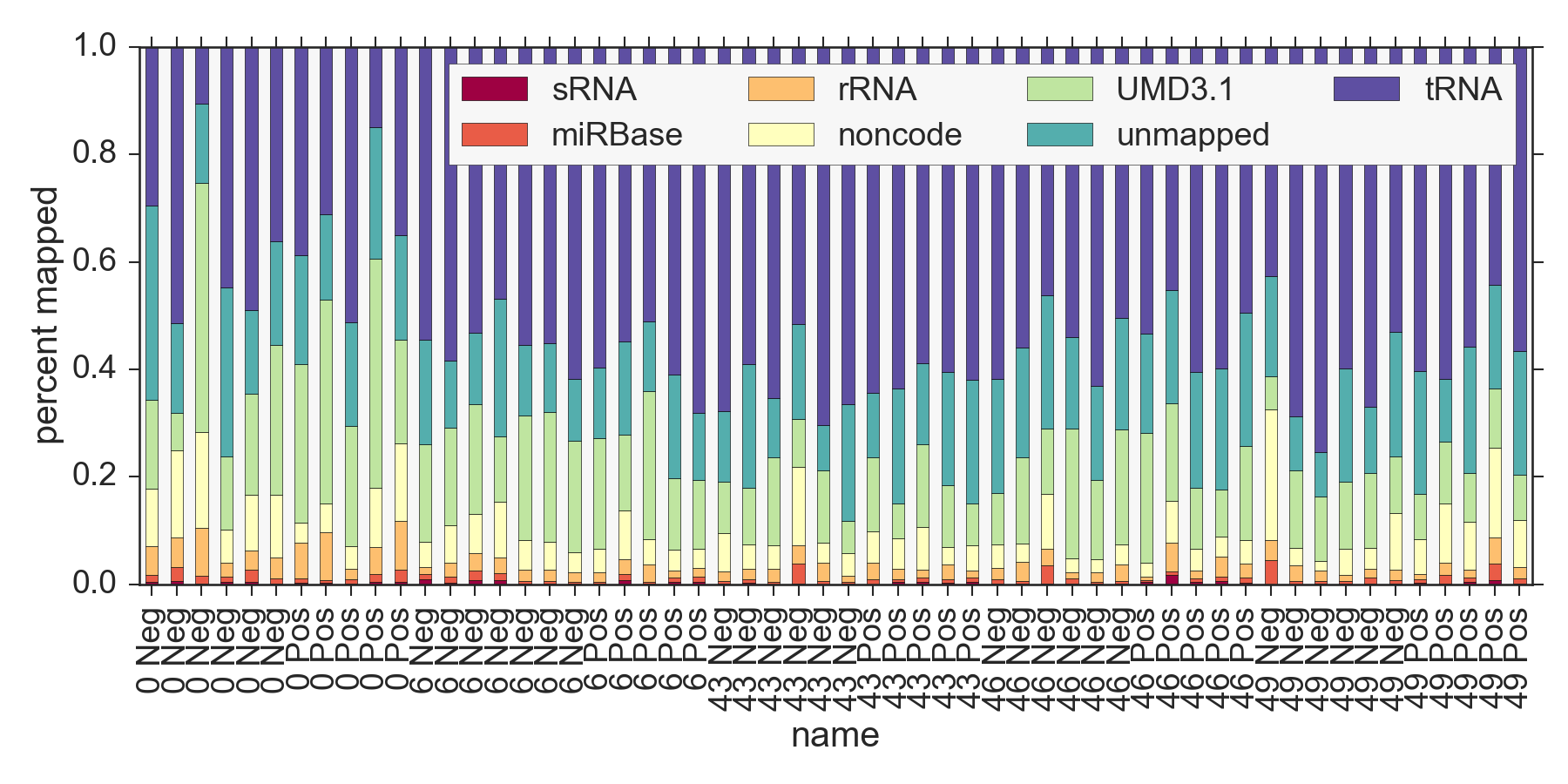

Supplement: S2 Fig — The samples are sorted by their timepoint (month) and ELISA status. (PNG) [file pone.0145089.s002.png]

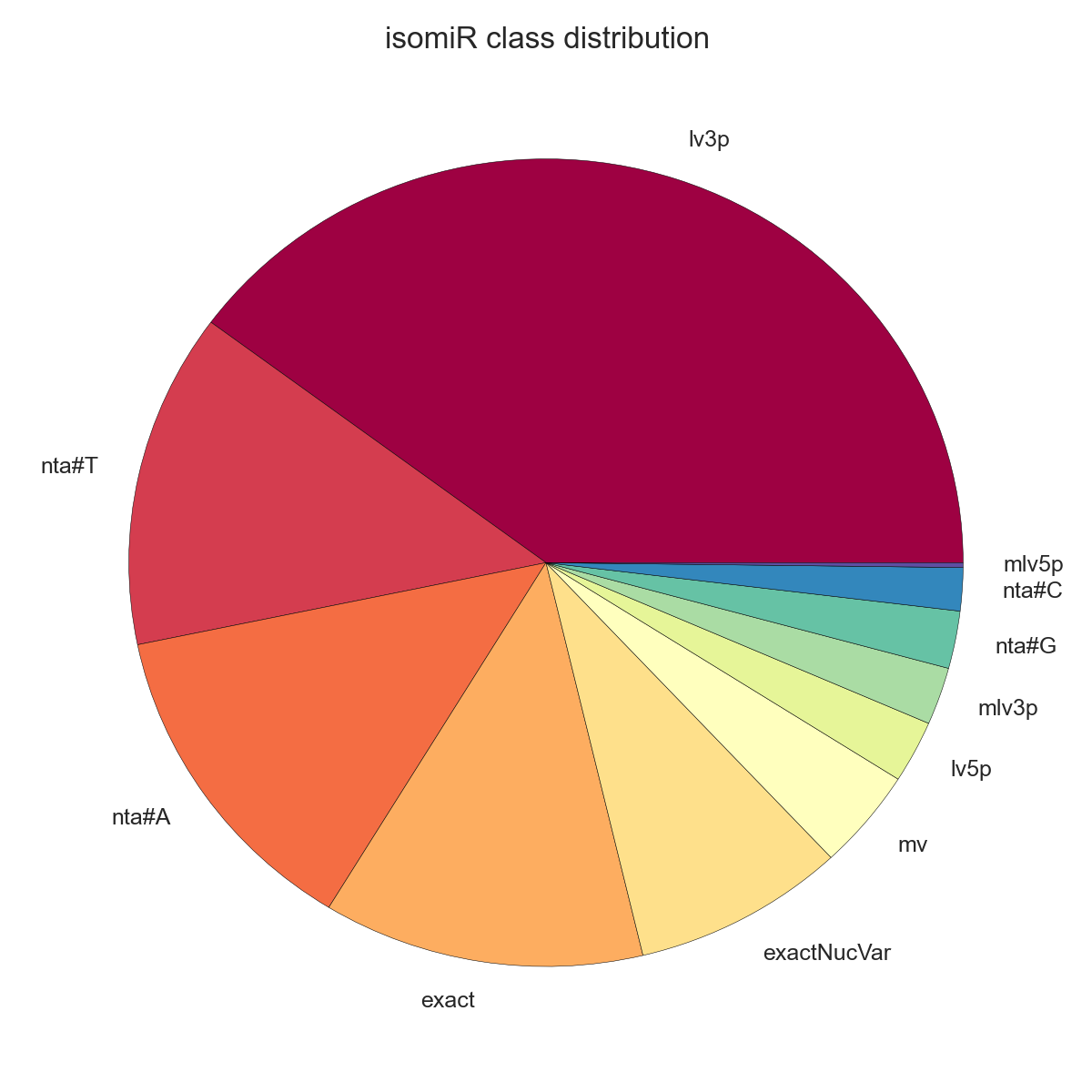

Supplement: S3 Fig — Labels for isomiR classes are using the sRNAbench hierarchical scheme. (PNG) [file pone.0145089.s003.png]

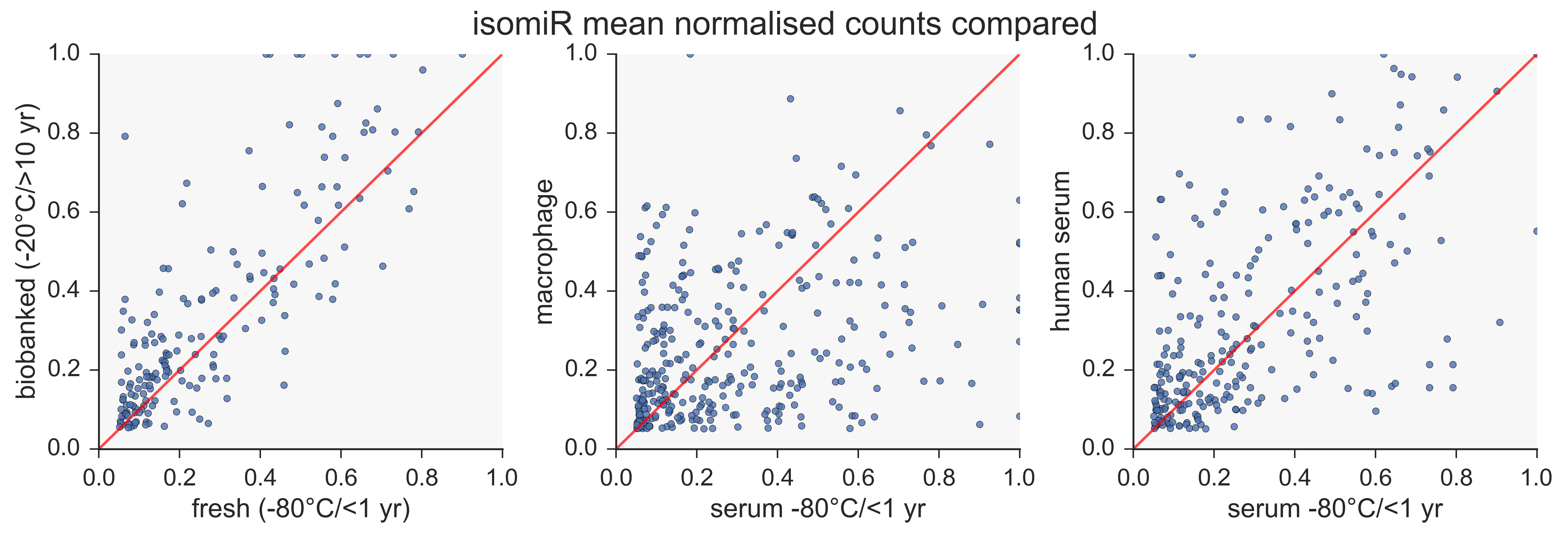

Supplement: S4 Fig — (PNG) [file pone.0145089.s004.png]

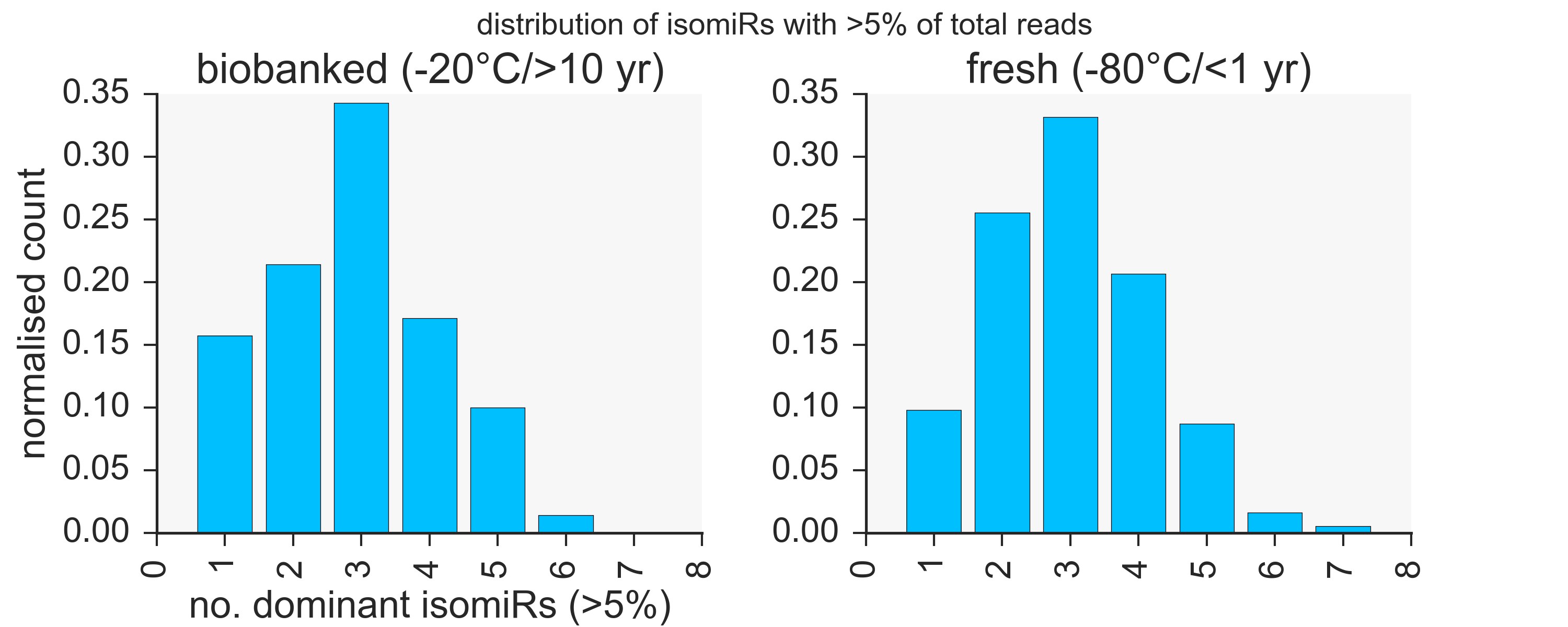

Supplement: S5 Fig — (PNG) [file pone.0145089.s005.png]

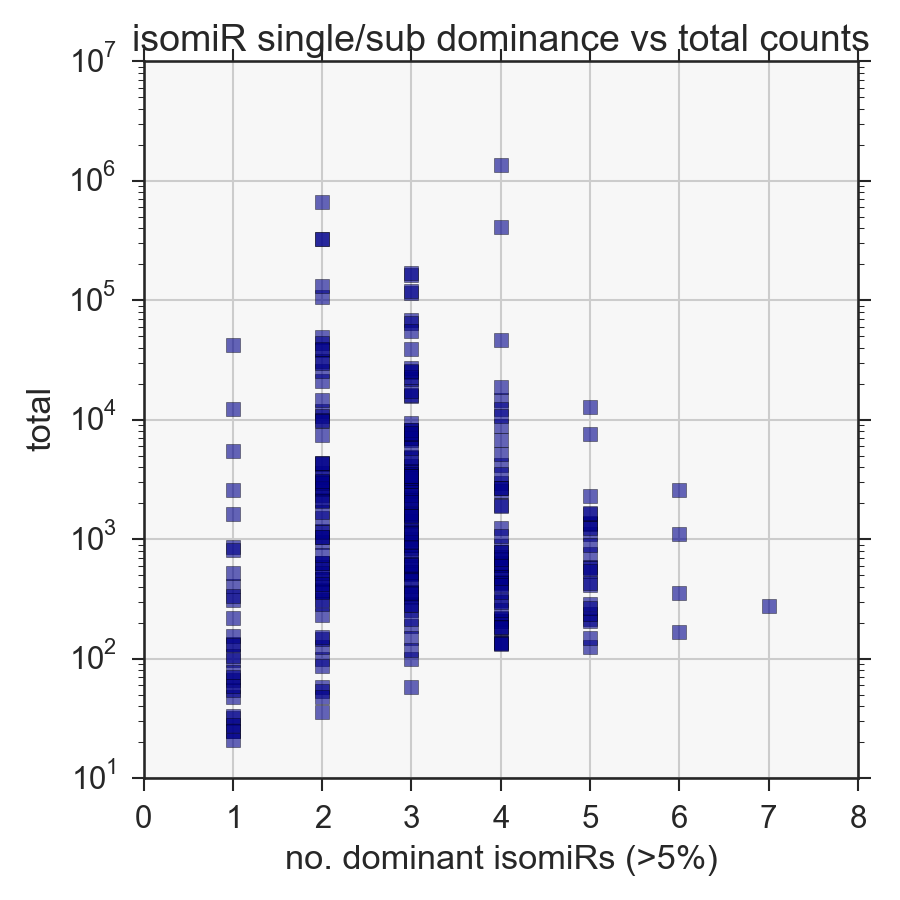

Supplement: S6 Fig — (PNG) [file pone.0145089.s006.png]
